# Is There a Correlation Between Masticatory Muscle Thickness and Pain After Botulinum Toxin Injections in Myogenic TMD Patients?: A Pilot Study

**DOI:** 10.3390/toxins17050220

**Published:** 2025-04-28

**Authors:** Hye-Ji Park, Hee-Jin Kim, Sung Ok Hong

**Affiliations:** 1 Department of Oral Medicine, Kyung Hee University Hospital at Gangdong, Seoul 05278, Republic of Korea; jibyji@gmail.com; 2 Division in Anatomy and Developmental Biology, Department of Oral Biology, Human Identification Research Institute, BK21 FOUR Project, Yonsei University College of Dentistry, Seoul 03722, Republic of Korea; hjk776@yuhs.ac; 3 Department of Oral and Maxillofacial Surgery, Kyung Hee University Hospital at Gangdong, Seoul 05278, Republic of Korea

**Keywords:** botulinum toxin, ultrasonography, temporomandibular disorder, masseter muscle, temporalis muscle, muscle thickness, myofascial pain

## Abstract

Botulinum toxin type A (BoNT-A), a potent neurotoxin, is increasingly used to treat myogenic temporomandibular disorders (TMDs); however, the interplay between muscle atrophy and pain relief remains incompletely understood. This pilot study investigated how masseter and temporalis muscle thickness and pain intensity change over 12 weeks following BoNT-A injections in 15 patients (mean age 51.42 years) with myogenic TMD. Muscle thickness was measured via ultrasonography across multiple anatomical positions under both clenching and resting conditions at baseline and at 2, 4, 8, and 12 weeks post-injection. Significant thinning of both muscles occurred within 2 weeks, lasting until 12 weeks, but became less pronounced after the first month. Pain intensity showed parallel decreases, most notably early on, but these reductions were not consistently statistically significant. Correlation analyses revealed no strong persistent association between muscle thickness and pain except for a moderately positive correlation in the anterior temporalis at two weeks (r = 0.61, *p* = 0.04). BoNT-A induces rapid masticatory muscle atrophy and modest pain relief; however, these outcomes do not coincide. Pain relief was observed earlier than the full development of muscle atrophy and should be considered during TMD pain management.

## 1. Introduction

Temporomandibular disorders (TMDs) are a group of musculoskeletal conditions affecting the temporomandibular joint, associated musculature, and surrounding structures [1]. Symptoms include pain during chewing, restricted mouth opening, and occasionally referred symptoms such as tinnitus and ear discomfort [2]. The diagnostic criteria for TMD (DC/TMD) classify these disorders into myogenous, arthrogenous, and mixed types, based on the primary source of dysfunction [3]. Of these subtypes, **myogenous TMD** is defined by pain originating in the masticatory muscles, which is exacerbated by jaw movement, function, or parafunction and reproduced upon muscle palpation. Because muscle hyperactivity or dysfunction is central to myogenous TMD, interventions targeting the involved musculature have become a key focus of clinical management [1].

Botulinum toxin type A (BoNT-A), a neurotoxin derived from *Clostridium botulinum*, inhibits the release of acetylcholine at neuromuscular junctions, resulting in temporary muscle paralysis [4,5]. Its clinical applications have expanded beyond cosmetic procedures to treat migraines, spasticity, and chronic pain [5,6]. Recent evidence suggests that BoNT-A exerts pain-modulating effects through multiple mechanisms, including reducing muscle nociceptor sensitivity, altering central pain processing, and decreasing neurogenic inflammation [5,7,8,9]. Additionally, BoNT-A injections can induce **muscle atrophy**, a reduction in muscle mass and volume resulting from decreased neuromuscular activity. This atrophic effect is utilized therapeutically in conditions such as masseteric hypertrophy, where excessive muscle bulk contributes to clinical symptoms [10,11]. BoNT-A has emerged as a promising therapeutic option for TMD, particularly in cases where conservative treatments, such as occlusal splints and nonsteroidal anti-inflammatory drugs (NSAIDs), provide limited relief [10,12]. Since the primary muscles where most patients with TMD experience discomfort are the masseter and temporalis muscles, these muscles are targeted solely or in combination using BoNT-A [13,14].

While previous studies have often examined the effects of botulinum toxin on pain or volume reduction individually, the relationship between muscle volume reduction and pain relief has not been thoroughly investigated, making it unclear whether these outcomes co-occur or follow distinct trajectories over time. In addition, no study has investigated the serial timing of the immediate effects of this treatment on pain in patients with myofascial TMD.

Ultrasonography (US) is increasingly being used in clinical research to accurately assess the effects of BoNT-A on masticatory muscle thickness [15]. US is a non-invasive, real-time imaging tool that accurately evaluates masticatory muscle thickness and structural changes following interventions such as BoNT-A injection [15,16]. Unlike other imaging modalities such as magnetic resonance imaging (MRI) or computed tomography, US provides high-resolution soft tissue visualization without radiation exposure, making it a practical choice for monitoring muscle adaptations over time [11]. Studies have shown that US effectively measures muscle thickness and functional changes, aiding in both diagnosis and treatment planning for TMD-related muscle hypertrophy [10].

This study aimed to examine time-dependent changes in masseter and temporalis muscle thicknesses following BoNT-A injection in patients with TMD, along with associated pain perception changes over three months. By analyzing volumetric changes across multiple anatomical positions under both clenching and resting conditions, this study sought to provide a comprehensive understanding of the effects of BoNT-A on the masticatory muscles and its implications for clinical practice in TMD management.

## 2. Results

This study included a total of 15 patients with a mean age of 51.42 ± 20.18 years. The sample comprised 4 males and 11 females. At baseline, the patients’ VAS score was 2.27 ± 2.73, and the maximum mouth opening was 42 ± 4.1mm.

### 2.1. Changes in Masticatory Muscle Thickness After BoNT-A Injection

**Figure 1:**
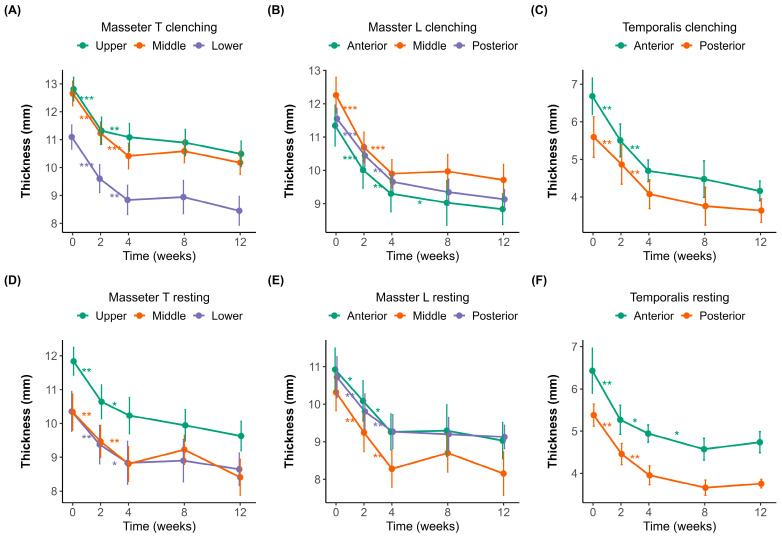
Changes in muscle thickness over 12 weeks after botulinum toxin injection. (**A**–**C**) Muscle thickness measured during clenching. (**D**–**F**) Muscle thickness measured during resting. (**A**,**D**) Transverse sections of the masseter muscle (Masseter_T) measured in the upper (purple), middle (orange), and lower (green) regions. (**B**,**E**) Longitudinal sections of the masseter muscle (Masseter_L) measured at the anterior (green), middle (orange), and posterior (purple) regions. (**C**,**F**) Temporalis muscle thickness at the anterior (green) and posterior (orange) regions. Error bars indicate standard errors. A significant reduction in muscle thickness was observed following botulinum toxin injection, with a plateau effect occurring around week 4. Statistical significance: *p* < 0.05 *, *p* < 0.01 **, *p* < 0.001 ***.

Muscle thickness was measured at different time points (0, 2, 4, 8, and 12 weeks) under both clenching and resting conditions (Figure 1A–F). The initial thicknesses of the masseter and temporalis muscles were measured using a probe in both the transverse (upper, middle, and lower; Figure 1A,D) and longitudinal (posterior, middle, and anterior; Figure 1B,E) orientations.

In the masseter muscle, the baseline thickness in the transverse orientation (Figure 1A,D) was 12.81 ± 1.59 mm and 11.84 ± 1.52 mm in the upper region, 12.66 ± 1.69 mm and 10.34 ± 2.06 mm in the middle region, and 11.10 ± 1.71 mm and 10.35 ± 2.27 mm in the lower region during clenching and resting, respectively. In the longitudinal orientation (Figure 1B,E), the corresponding baseline values were 11.55 ± 1.15 mm and 10.72 ± 2.03 mm in the posterior region, 12.26 ± 2.09 mm and 10.31 ± 1.89 mm in the middle region, and 11.34 ± 2.37 mm and 10.92 ± 2.20 mm in the anterior region during clenching and resting, respectively.

Significant reductions in masseter muscle thickness were observed following BoNT-A injections, most notably within the first two weeks. In the transverse sections (Figure 1A,D), the upper region experienced the most significant initial decline, 1.52 ± 0.87 mm (*p* < 0.001), indicative of substantial early muscle atrophy. Similarly pronounced decreases were noted in the middle (1.40 ± 0.72 mm, *p* < 0.001) and lower regions (1.17 ± 0.56 mm, *p* < 0.001), suggesting consistent atrophy across different transverse regions initially. Between weeks 2 and 4, while the thickness continued to decrease, the magnitude of changes diminished significantly (middle region, 1.04 ± 0.71 mm, *p* < 0.01), pointing toward slowing atrophic processes. After week four, the reduction slowed dramatically, suggesting that the muscle reached near-maximal atrophy during this period.

The longitudinal sections of the masseter (Figure 1B,E) demonstrated similar patterns, with initial significant reductions across anterior (1.25 ± 0.73 mm, *p* < 0.001), middle (1.40 ± 0.83 mm, *p* < 0.001), and posterior (1.14 ± 0.41 mm, *p* < 0.001) regions. The most significant muscle thinning occurred early, consistent with the transverse findings, followed by incremental but significantly reduced atrophy after week four.

The temporalis muscle (Figure 1C,F) displayed an analogous pattern, with the anterior and posterior regions experiencing notable initial decreases of 1.14 ± 1.52 mm (*p* < 0.01) and 0.97 ± 0.60 mm (*p* < 0.01), respectively. The initial thickness was 6.68 ± 1.95 mm during clenching and 6.43 ± 2.13 mm during resting in the anterior region, and 5.60 ± 2.02 mm during clenching and 5.38 ± 0.98 mm during resting in the posterior region. Regardless of posture, muscle thickness decreased until week 8. After week 8, the muscle thickness slightly increased during clenching compared to the previous measurement, whereas it slightly decreased at rest. However, the changes observed after week eight were not statistically significant.

### 2.2. Changes in Difference Between Clenching and Resting Conditions

**Figure 2:**
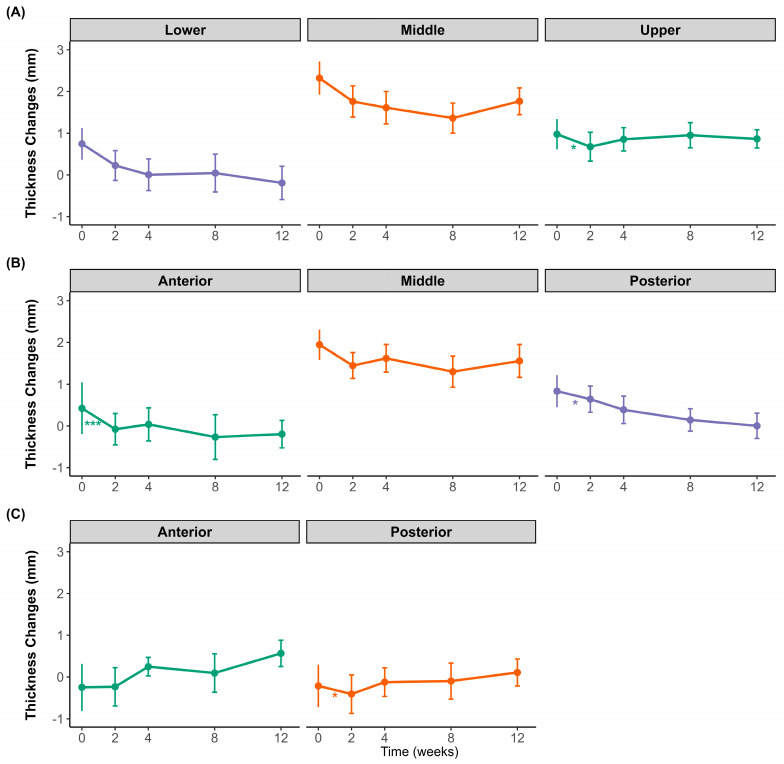
Changes in muscle thickness difference between clenching and resting over 12 weeks after botulinum toxin injection. (**A**) Transverse sections of the masseter at the upper (purple), middle (orange), and lower (green) regions. (**B**) Longitudinal sections of the masseter at the anterior (green), middle (orange), and posterior (purple) regions. (**C**) Temporalis muscle thickness at the anterior (green) and posterior (orange) regions. Data points show mean values with standard deviations. * *p* < 0.05, *** *p* < 0.001; ns, not significant.

Figure 2 illustrates the changes in the difference between clenching and resting muscle thicknesses in the masseter and temporalis muscles over 12 weeks following BoNT-A injection. Regional differences were observed in the masseter muscle. The lower and posterior regions showed an overall decreasing trend throughout the observation period, whereas the middle region of the masseter muscle initially decreased until week 4 and then slightly rebounded at weeks 8 and 12. The upper region also showed an initial decline at week 2, followed by a gradual increase toward week 12. In the anterior and middle regions, the difference in clenching–resting thickness initially decreased at week 2, followed by a partial recovery at subsequent time points. However, this initial decrease was only statistically significant in the middle region. For the temporalis muscle, both the anterior and posterior regions exhibited minor fluctuations and a subtle increase toward the end of the study period; however, these changes were not statistically significant.

### 2.3. Pain Intensity of Masticatory Muscle After BoNT-A Injection

The changes in pain intensity in each muscle region after BoNT-A injection are summarized in Table 1. The overall pain intensity decreased from baseline (2.27 ± 2.73) to 2 weeks (1.40 ± 1.70), although this change was not statistically significant (*p* = 0.39). No statistically significant differences were observed between subsequent time points (2–4 weeks, *p* = 0.78; 4–8 weeks, *p* = 0.39; 8–12 weeks, *p* = 0.92).

For the masseter muscle, pain intensity at both upper and lower measurement points showed a tendency to decrease from baseline to 2 weeks (upper masseter: 2.85 ± 1.65 to 1.81 ± 1.06; lower masseter: 2.89 ± 1.69 to 1.77 ± 1.24), but these differences approached only borderline statistical significance (both *p* = 0.065).

Regarding the temporalis muscle, pain intensity at the anterior measurement point exhibited a marked decrease from baseline (2.44 ± 1.59) to 2 weeks (1.37 ± 1.11), which was also marginally above statistical significance (*p* = 0.056). Similarly, the posterior temporalis measurement showed a considerable decrease from baseline (2.01 ± 1.53) to 2 weeks (0.93 ± 1.15) with borderline statistical significance (*p* = 0.052).

Collectively, pain intensity tended to decrease after BoNT-A injection across all evaluated regions, especially within the first 2 weeks. However, these reductions were not statistically significant at the conventional threshold of *p* < 0.05.

### 2.4. Changes and Correlations in Muscle Thickness and Pain Intensity

**Figure 3:**
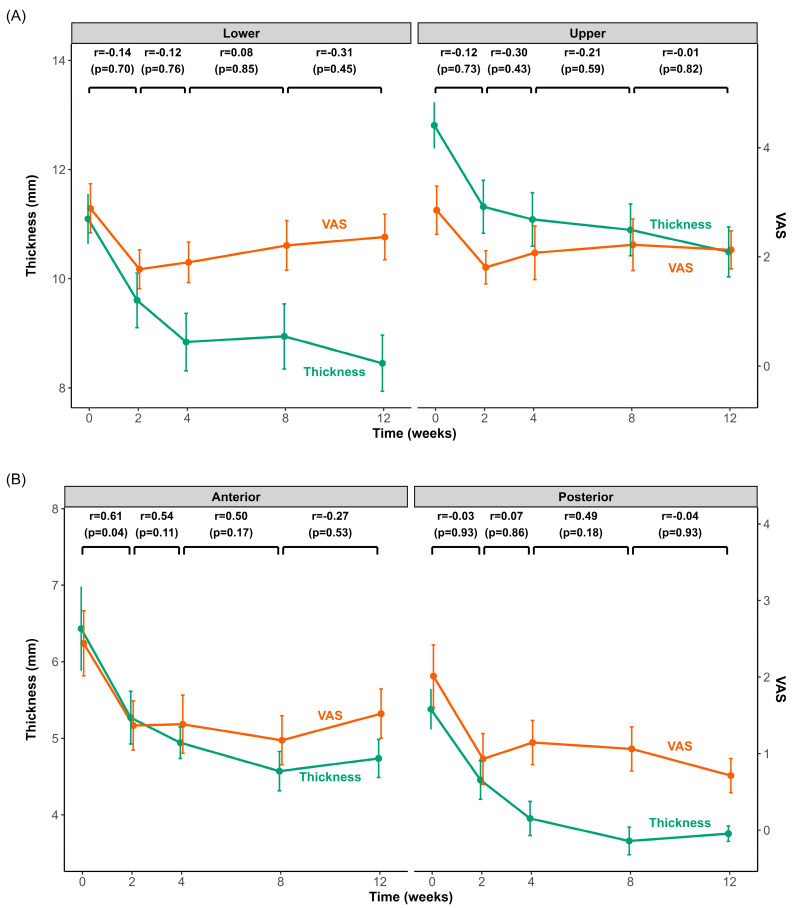
Changes in muscle thickness and pain intensity after BoNT-A injection in the masseter and temporalis muscles over 3 months. Time-course changes in muscle thickness (green line) and pain intensity (visual analog scale (VAS), orange line) for the masseter (**A**) and temporalis (**B**) muscles from baseline (0 weeks) to 12 weeks after BoNT-A injection. The left y-axis shows muscle thickness (mm), and the right y-axis shows the VAS score. Each data point represents the mean ± standard error of the mean (SEM). Pearson’s correlation coefficients (r) and *p*-values above the graphs indicate the correlations between the changes (Δ) in muscle thickness and VAS across consecutive intervals (0–2 weeks, 2–4 weeks, 4–8 weeks, and 8–12 weeks).

Figure 3 illustrates the time-course of changes in muscle thickness and pain intensity following BoNT-A injection into the masseter (upper and lower regions) and temporalis (anterior and posterior regions) muscles. Both muscles exhibited significantly reduced thickness and pain intensity within the initial 2 weeks post-injection. After this initial rapid decline, the rate of change slowed significantly, with muscle thickness continuing to decrease slightly until approximately 4 weeks and then stabilizing or showing minimal variation thereafter. Pain intensity in the masseter and temporalis reached its lowest point at 2 weeks and increased slightly afterwards.

Correlation analyses revealed no statistically significant relationship between muscle thickness and pain intensity at any evaluated interval for the masseter muscle. However, a significant correlation was observed in the temporalis muscle, specifically in the anterior region, during the initial 2-week period (r = 0.61, *p* = 0.04). No other intervals exhibited significant correlations.

**Figure 4:**
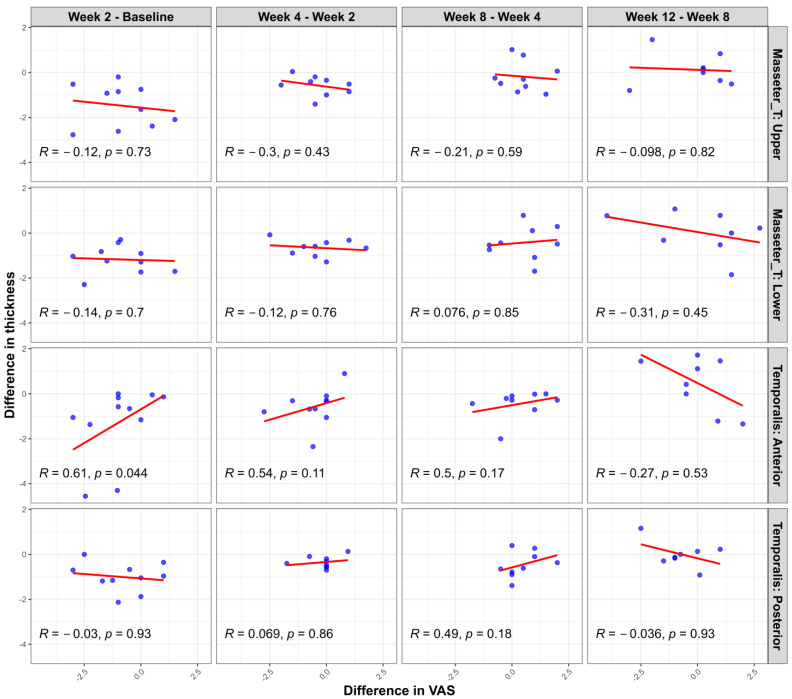
Correlation between changes in muscle thickness difference (clenching–rest) and pain intensity (VAS) changes at consecutive measurement intervals following BoNT-A injection. Scatter plots illustrate the relationship between interval changes in muscle thickness (measured during clenching versus rest conditions) and interval changes in pain intensity (VAS) in each muscle region (masseter upper, masseter lower, temporalis anterior, and temporalis posterior). Pearson’s correlation coefficients (R) and corresponding *p*-values are provided in each panel. A statistically significant correlation was observed only in the temporalis anterior region between the first and second evaluations (R = 0.65, *p* = 0.03). The other areas and intervals demonstrated no significant correlations.

Additional scatter plot analyses (Figure 4) evaluating the changes in muscle thickness between the clenching and rest conditions at each measurement time point relative to changes in pain intensity (assessed with a visual analog scale (VAS)) revealed limited significant correlations. Specifically, a statistically significant positive correlation was noted only in the temporalis anterior region during the initial evaluation interval (1st–2nd measurement; r = 0.65, *p* = 0.03). Other muscle regions and subsequent intervals showed no significant correlations between muscle thickness and VAS changes. These findings further support the observation that, while showing similar overall trends, muscle thickness and pain intensity changes do not consistently demonstrate strong direct relationships at the individual patient level.

## 3. Discussion

The positive effects of BoNT-A on several chronic pain conditions has been reported through various clinical trials and reviews [5,17,18,19,20]. A recent systematic review and meta-analysis of 15 randomized controlled trials (RCTs) found that BoNT-A was associated with a significant reduction in pain intensity at 1, 2, 3, and 6 months compared to placebo [21]. This study observed a possible relationship between the VAS and muscle thickness change after botulinum toxin injection in patients with myofascial pain. This is the first study to evaluate changes in muscle thickness between the resting and clenching states at different time points on several different points in both the masseter and temporalis muscles.

US is a cost-effective, non-invasive, quick (average 10–15 min) diagnostic tool that can be easily used in the clinical setting and has many advantages over MRI [22]. Real-time assessment can be performed without sedation for children or older adults, and studies have highlighted its potential for diagnosing or treating masticatory muscle pain [23,24]. Muscle thickness measurements using US have been implemented in many cases in the oral and maxillofacial area [25,26]. A comparative study between MRI and US found significant correlations, thus suggesting that both methods are reliable for assessing masseter muscle thickness [27].

The average thickness of masticatory muscles varies by sex and age [22,28]. A Korean study found that the masseter muscle thickness in healthy Korean adults was 9.8 ± 1.3 mm and 12.4 ± 1.4 mm in females and 11.3 ± 1.2 mm and 14.7 ± 1.4 mm in males at resting and clenching, respectively [29]. In the present study, the average masseter muscle thickness was 12.81 ± 1.59 mm and 11.84 ± 1.52 mm in the upper region, 12.66 ± 1.69 mm and 10.34 ± 2.06 mm in the middle region, and 11.10 ± 1.71 mm and 10.35 ± 2.27 mm in the lower region during clenching and resting, respectively. For the temporalis muscle, it was 6.43 ± 2.13 mm during resting and 6.68 ± 1.95 mm during clenching in the anterior region and 5.38 ± 0.98 mm during resting and 5.60 ± 2.02 mm during clenching in the posterior region. Such differences can be explained by the lack of standardization of reference points during measurement, different ages, patient positioning, and relaxed versus contracted muscles [22,30].

Despite known differences in average muscle thickness between men and women, we could not perform sex-specific analyses due to the limited sample size. However, to account for these differences when comparing changes, we conducted an additional analysis by calculating Z-scores based on sex-specific means and standard deviations (Tables S1 and S2). Notably, the findings from this supplementary analysis were consistent with our original results, suggesting that our overall conclusions remain robust.

In the present study, we looked at several different anatomical positions on the masseter and temporalis muscles [11]. There is literature sectioning the masseter muscles into one-third sections and looking at the anatomy, but most of the literature looks at only one point on the masseter muscles [11,22]. The anterior temporalis landmark is defined as the area located on the temple level of the pupil or in front of the anterior border of the hairline [28,31]. The temporalis muscle can be divided into two functional parts: anterior and posterior. The anterior fibers run vertically, elevating the mandible, while the posterior fibers run horizontally, retruding the mandible [32]. Interestingly, a significant reduction in masseter muscle thickness was observed within the first two weeks, regardless of the injection area. The upper transverse third of the masseter muscle decreased more significantly than the middle and lower masseter areas, where BoNT-A was directly injected. Although the middle masseter muscle reduced the most in the longitudinal section, the anterior section also decreased more than the posterior section, where direct injection had been performed. For the temporalis muscle, a significant reduction was observed in the first two weeks, but this reduction was more significant in the anterior region. Simultaneous injection of BoNT-A into the masseter and temporalis muscles resulted in muscle reduction. In previous studies, injection into only the masseter muscles increased compensatory muscle thickness in the temporalis muscles, which caused pain after 6 months [12]. A simultaneous muscle thickness reduction was observed in studies where BoNT-A was injected into both masticatory muscles [20]. In previous research, changes in masseter muscle thickness following botulinum toxin injection were more strongly influenced by initial muscle thickness than by age. When muscle thickness changes were compared across different age groups (10s, 20s, 30s, 40s, and 50s), no significant differences were observed, except in the 40-year-old group, which had the greatest baseline muscle thickness [33]. BoNT-A binds to presynaptic receptors such as SV2 and synaptotagmin and cleaves SNAP-25, which is a critical component of the SNARE complex required for vesicle function. Cleavage prevents exocytosis of acetylcholine, causing flaccid paralysis of the muscles. The motor effect persists until new SNARE proteins are synthesized and new neuromuscular junctions are reformed [34]. Muscle thickness and occlusal force typically decrease until three months and start to recover afterwards [12,35,36]. This is similar to the present study, where a significant reduction in muscle thickness compared to baseline was seen in all sections of the masseter and temporalis muscles. Although the temporalis muscle showed functional recovery during clenching after two months, this was not significant.

Several systematic reviews have evaluated the effect of BoNT-A therapy for myofascial pain [21,35,37,38]. A recent review found that BoNT-A caused a significant reduction in pain intensity at 1, 2, 3, and 6 months post-treatment compared to placebo [21]. The mechanisms of action are not fully understood, but studies suggest the analgesic effect involves a reduction in neurotransmitters and neuromodulators, such as substance P, calcitonin gene-related peptides, and glutamate released from sensory nerve endings [38,39]. This leads to decreased neurogenic inflammation and peripheral sensitization. Retrograde transport, which affects central pain processing, may mitigate this process. As a result, patients may experience analgesic effects sooner than muscular atrophic changes. Ranoux et al. [40]. observed reduced pain intensity at two weeks in patients with allodynia. A randomized crossover trial by Sitnikova et al. [36] demonstrated that the muscle-relaxing effect of the toxin was not responsible for a reduction in TMD-related myofascial pain syndrome. After 50 U of BoNT-A, 42%, 30%, and 32% of the patients achieved a significant pain reduction (≥30%) at 2, 11, and 16 weeks. This finding also correlates with another crossover RCT reporting 43% and 33% at 1 and 3 months [41]. Another recent study also observed a significant reduction in pain by 30% at 15 days post-injection, but the effect lasted only 30 days due to the small injection dose of 20 U [42]. The most important factor regulating the longevity of toxin action is the ability of BoNT-A protease to avoid cellular degradation and survive in the cell cytoplasm for a long period [5].

The effect of BoNT-A is related to localization and dosage; therefore, 25–50 U for the masseter muscles and 5–25 U for the temporalis muscles at five different injection sites have been preferred in previous studies [43,44]. In the present study, 30 U was administered to the masseter and 20 U to the temporalis muscles [45]. A recent study randomized 100 patients to three different doses of BoNT-A (low, medium, and high) [46]. The low dose was 30 U in the masseter and 10 U in the temporalis, the medium was 50 U in the masseter and 20 U in the temporalis, and the high dose was 75 U in the masseter and 25 U in the temporalis. No significant differences were found among the BoNT-A groups; however, a significant decrease in subjective pain was observed at 1 week, which lasted until the 24-week endpoint. This indicates a potential analgesic effect independent of the accompanying dose-dependent motor activity [47]. Montes-Carmona et al. also found a significant decrease in pain starting at 7 days after BoNT-A injection, proposing that the peak of pain effectiveness occurred due to the analgesic effect gradually accumulating after a week of continuous muscle relaxation [48]. In this study, pain intensity did not significantly decrease, but there was a consistent reduction in the lower region of the masseter muscle and the temporalis muscle, with a significant decrease in both muscles within the first 2 weeks. Injections of BoNT-A in the masseter, temporalis, and pterygoid muscles are linked to a greater reduction in pain, whereas masseter and temporalis injections have a lower effect size at 6 months [21]. The limited sample size and injection area might have contributed to these findings.

In previous studies, the baseline TMD pain VAS among symptomatic patients prior to botulinum toxin injection commonly ranged between four and seven [38]. By contrast, the mean total VAS in our study was relatively low (2.27 ± 2.73) and showed a large standard deviation. For instance, Pihut et al. [49] reported an average VAS of four, but individual scores ranged widely from two to eight, highlighting the inherent variability in pain perception. Our limited sample size may have further amplified this effect, as a small number of individual values could disproportionately influence the overall mean. Moreover, TMD-related pain is highly subjective: patients experiencing intermittent rather than persistent chronic pain may register lower baseline VAS scores. Nevertheless, the palpation VAS for the masseter and temporalis muscles was more stable than the total VAS, presumably because applying a standardized force (1 kgf) during palpation helps reduce subjective variation in pain reporting.

In this study, the maximum analgesic effect preceded the maximum muscle atrophic effect. The maximum pain reduction was observed at 2 weeks, whereas atrophy was observed at 12 weeks. A plausible explanation may be that sensory nerve endings are more sensitive to botulinum toxin; therefore, even a partial neurotransmitter release blockade can rapidly reduce pain signaling. Another hypothesis is that the molecular machinery in sensory synapses may have a faster turnover rate or a lower threshold for inhibition, leading to a quicker onset of analgesia. The discrepancy between pain and size change can also be explained by immediate functional inhibition of neurotransmitter release, which leads to analgesia. In contrast, muscle atrophy is a cumulative structural change resulting from prolonged disuse.

This study has several limitations. Because this was a cross-sectional study without a regular control group, only comparisons of the masticatory muscles within one TMD group were performed. The short follow-up period, wide age range, and small sample size may have influenced the results. Even though variables were compared by sex, the small number of patients was a limitation. Few studies have examined the impact of age on changes in masticatory muscle thickness after botulinum toxin injection. Due to the small sample size and wide age range, investigation of such factors was difficult. Future studies should consider long-term, larger sample sizes with different age and sex groups. Another limitation is that US is dependent on the equipment, operator, and the image interpreter, which can affect reproducibility and generalizability [50]. Real-time changes in US waves during examinations require experience, and even though both examiners showed high inter-rater reliability, there are still some limitations in standardizing measurements.

## 4. Conclusions

This study aimed to assess short-term US-based changes in the thickness of the masticatory muscles (masseter and temporalis) at various anatomical sites during the resting and clenching states in relation to the level of pain. BoNT-A showed a statistically significant change in the thickness of both the masseter and temporalis muscles at 2 weeks and 1 month, whereas pain showed significant reduction at 2 weeks. In conclusion, the motor and sensory effects after BoNT-A injection in patients with myofascial TMD occurred at different time points, showing a different relationship between TMD pain and muscle atrophy caused by botulinum toxin treatment. Pain relief was observed earlier than the full development of muscle atrophy. Treatment of TMD pain with botulinum toxin still requires a wide range of further research to understand the mechanism of botulinum toxin’s pain-relieving properties.

## 5. Materials and Methods

### 5.1. Study Design

This retrospective study aligned with the principles of the Declaration of Helsinki and was approved by the Institutional Review Board of Kyung Hee University Dental Hospital in Gangdong, Seoul, Republic of Korea (IRB number 2025-01-019).

This study included 68 patients who visited Gangdong Kyung Hee Dental Hospital for TMD treatment using botulinum toxin between January 2022 and August 2024. All patients underwent a thorough examination and diagnosis by two TMD specialists with more than 10 years of clinical experience. The patients were diagnosed with myogenic TMD of the masticatory muscles according to the DC/TMD criteria. Of the 68 patients, patients with VAS scores and US images at follow-up (2 weeks after injection, 1 month after injection, 2 months after injection, and 3 months after injection) were included. Patient inclusion criteria were (1) age over 18 years old, (2) myalgia of the masseter and/or temporalis muscle, (3) no history of TMJ sound, (4) no malocclusion, and (5) no mouth opening limitation. Demographics such as age, sex, maximum mouth opening, and general conditions were investigated.

### 5.2. Procedures and Assessment

**Figure 5:**
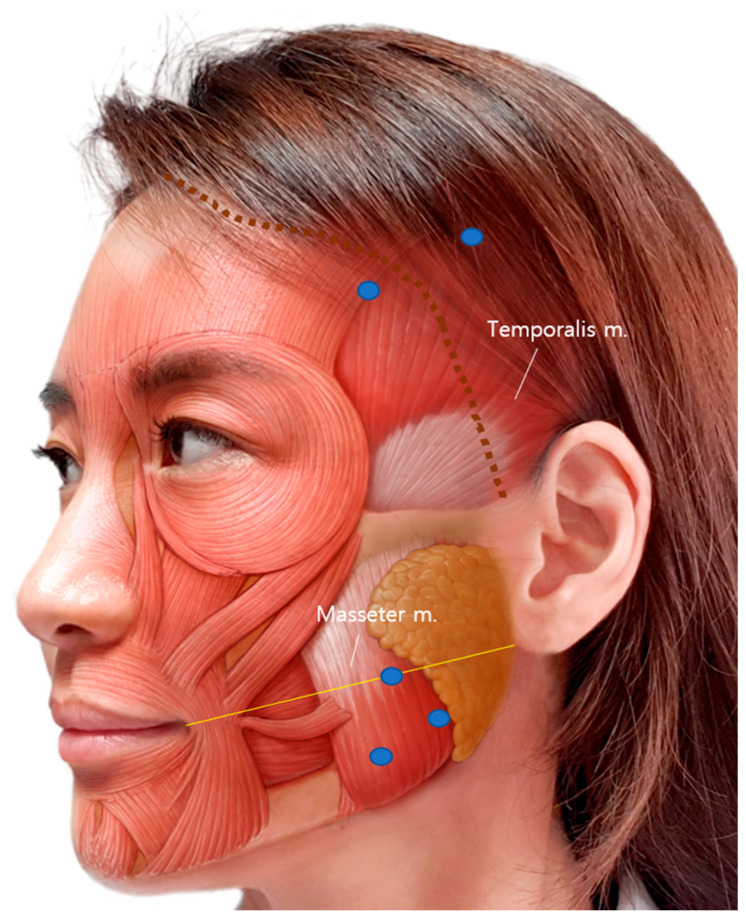
BoNT-A injection sites of the temporalis and masseter muscles. Three injections on the masseter and two injections on the temporalis were performed. The yellow line is a reference line connecting the cheilion to otobasion inferius. The blue dots indicate the injection points, and the dotted brown line indicates the actual hairline.

BoNT-A 100 U (LIZTOX^®^, Humedix, Seongnam-si, Republic of Korea) was mixed with 2 mL of 0.9% isotonic sterile saline solution and stored at room temperature. Bilateral intramuscular injection was performed using a 1 mL syringe with a 30-gauge and 13 mm needle. For each masseter muscle, 30 U was injected into three sites (10 U per site) at least 5 mm apart. The cheilion to otobasion inferius line was used as a reference line for the masseter muscle injection [25,51]. The most prominent point was checked after clenching and included in the injection points. For each temporalis muscle, 20 U was injected at two sites (10U per site): one point at the hairline level and one point behind the hairline, at least 5 mm apart (Figure 5).

A VAS, a 100 mm horizontal line marked with “no pain” at the left end and “worst pain imaginable” at the right end, was used to assess pain. When checking selected points on the masseter and temporalis muscles, the participants were asked to mark a line pointing to their present pain level. The overall pain level felt during within the last month without palpation of any muscles was recorded as total VAS. Muscle pressure was assessed by using a digital algometer (Wagner FPX^TM^, Greenwich, CT, USA). The algometer had a flat, circular tip with an area of 1 cm^2^ positioned perpendicularly to the muscles, applying a pressure of approximately 98 kPa (equivalent to 1.0 kgf/cm^2^). Pressure was applied to the upper and lower halves of the masseter and anterior and posterior temporalis muscles.

Subjective pain intensity, assessed by the VAS, and muscle thickness measurements using US were checked at five points: before treatment (baseline), 2 weeks after injection, 1 month after injection, 2 months after injection, and 3 months after injection. Patients who were assessed at least four times were included in this study.

Patients who had previously undergone aesthetic/therapeutic procedures such as BoNT-A injections, facial collagen or fillers in the face, maxillomandibular jaw surgery, and facial soft tissue surgery within 6 months were excluded from this study. Patients with autoimmune or neuromuscular diseases, those allergic to BoNT, pregnant or breastfeeding women, and those taking analgesic medications were excluded from this study.

**Figure 6:**
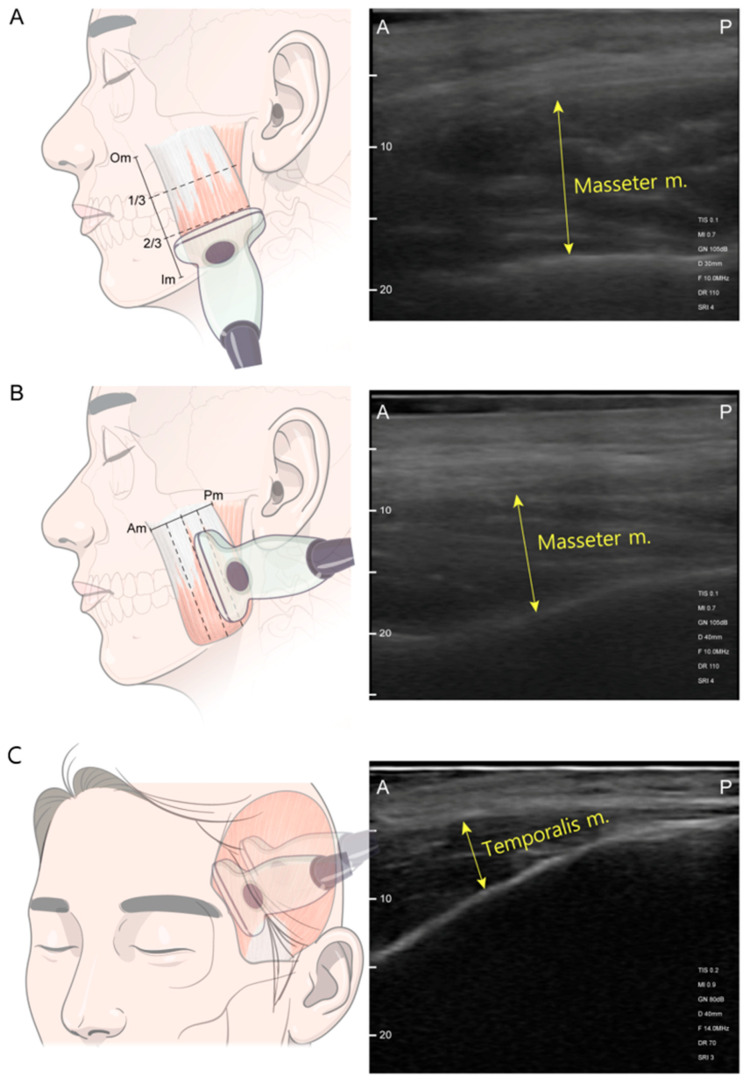
Ultrasonography scanning sites. (**A**) Transverse section scanning was performed at the upper-, mid-, and lower-third level between the masseter muscle origin (Om) and the masseter muscle insertion (Im) along the black dashed line. (**B**) Longitudinal section scanning was performed along the blue dashed line passing through the mesial, mid-, and distal points between the anterior border of the masseter muscle (Am) and the posterior border of the masseter muscle (Pm). (**C**) Temporalis muscle scanning at anterior and posterior points of the hairline. Abbreviations: 1/3, one-third level between the Om and Im; 2/3, lower-third level between the Om and Im; A, anterior; P, posterior.

US was performed using a SONO-Finder ultrasound system (N-Finders Co., Ltd., Seoul, Republic of Korea) paired with a linear transducer operating in the pulse frequency range of 10–14 Mhz. The patients were positioned upright in a head-at-rest seated position at a consistent height, which favored the examination. The facial area and body were relaxed, with the lips gently brought together. The US transducer was placed perpendicular to the skin surface of the masticatory muscle with thick US gel application and minimal mechanical pressure during evaluation. Muscle thickness was measured in both resting and clenching states of the masseter and temporalis muscles. For clenching measurements, patients were first asked to bite as forcefully as possible in their habitual bite position for 3 s. For resting measurements, patients sequentially relaxed their bite, slightly keeping their molar teeth apart [31]. Masseter muscle measurements were performed on three horizontal and vertical axes, including the most prominent point (Figure 6). Reference lines for the longitudinal axes of the masseter were (1) the anterior border of the muscle, (2) posterior border of the muscle, (3) halfway midline between the borders, (4) anterior halfway of the midline, and (5) posterior hallway of the midline. Reference lines of the transverse axes were (1) the line below the zygomatic arch, (2) inferior margin of the mandible, (3) halfway midline between the zygoma and mandible border, (4) upper halfway of the midline, and (5) lower halfway of the midline [11].

Image acquisition was carried out twice, first by capturing the images in real time, and second by capturing video in real-time. The thickness of the masseter and temporalis muscles were measured at the thickest point within the image [28,31,51]. The transducer was positioned parallel to the long axis of the zygomatic arch on the horizontal axis, and the probe was positioned perpendicular to the zygomatic arch on the vertical axis (Figure 6). A linear probe was positioned parallel to the outer edge of the eyebrow for the temporalis muscle, maintaining a distance of at least 2 cm from the orbital rim [11,50]. One point anterior to the hairline and one point posterior to the hairline were measured perpendicular to the temporal bone [28]. Thick US gel application and minimal mechanical pressure during evaluation allowed minimal voids when measuring the thickness of the posterior temporalis muscle. Muscle thickness was defined as the distance between the outer and inner fasciae. Measurements were recorded in millimeters, representing the average between both sides of each measurement point.

### 5.3. Statistical Analysis

Continuous variables are presented as means and standard deviations (SDs). Comparisons between the two time points were performed using the Wilcoxon signed-rank test. The relationship between two outcome measures was evaluated for each consecutive time interval using Spearman’s rank correlation analysis. For overall comparisons across all time points, the Kruskal–Wallis test was used, while pairwise comparisons between specific time points were performed using the Wilcoxon signed-rank test. All tests were two-sided, and *p*-values less than 0.05 were considered statistically significant. Statistical analyses were performed using R version 4.2.3. Inter- and intra-observer reliabilities were used to assess the degree of agreement among multiple repetitive measurements. Two investigators measured all the parameters on the ultrasound scans twice (H.J.P. and S.o.H.). Intraclass correlation coefficients (ICCs) were computed, using a threshold of 0.80 as the minimum acceptable reliability level for all measured items. The intra-examiner reproducibility demonstrated ICC values of 0.84 and 0.92. Items with ICCs below the established threshold were flagged for further evaluation and remeasured. Discrepancies were resolved through multiple discussions until an agreement was reached. All re-evaluated items consistently satisfied the predetermined ICC standard (>0.80).

## Figures and Tables

**Table 1:** Changes in pain intensity of masseter and temporalis muscles at different time points up to 3 months after BoNT-A injection.

Time										
		Baseline	2 Weeks		1 Month		2 Months		3 Months	
		Mean (SD)	Mean (SD)	*p*	Mean (SD)	*p*	Mean (SD)	*p*	Mean (SD)	*p*
Total VAS		2.27 (±2.73)	1.40 (±1.70)	0.058	1.63 (±1.97)	0.208	0.94 (±1.43)	0.361	1.00 (±0.84)	1.000
Masseter	Upper	2.85 (±1.65)	1.81 (±1.06)	0.084	2.07 (±1.84)	0.234	2.22 (±1.63)	0.634	2.13 (±1.15)	0.905
	Lower	2.89 (±1.69)	1.77 (±1.24)	0.068	1.90 (±1.39)	0.260	2.21 (±1.58)	0.504	2.36 (±1.38)	0.720
Temporalis	Anterior	2.44 (±1.59)	1.37 (±1.11)	0.031	1.38 (±1.42)	0.150	1.18 (±1.11)	0.944	1.52 (±1.06)	0.553
	Posterior	2.01 (±1.53)	0.93 (±1.15)	0.057	1.15 (±1.08)	0.584	1.06 (±1.00)	0.598	0.71 (±0.74)	0.288

## Data Availability

The data presented in this study are available on request from the corresponding author (the data are not publicly available due to privacy restrictions).

## Supplementary Materials

The following supporting information can be downloaded at: https://www.mdpi.com/article/10.3390/toxins17050220/s1, Table S1: Z-score of Figure 1; Table S2: Z-score of Figure 2.

## Abbreviations

The following abbreviations are used in this manuscript:

BoNT-A	Botulinum toxin type A
DC/TMD	Diagnostic criteria for temporomandibular disorders
MRI	Magnetic resonance imaging
TMD	Temporomandibular disorders
US	Ultrasonography
VAS	Visual analog scale
